# Antisense Oligonucleotide Technologies to Combat Obesity and Fatty Liver Disease

**DOI:** 10.3389/fphys.2022.839471

**Published:** 2022-01-28

**Authors:** Michael F. Keating, Brian G. Drew, Anna C. Calkin

**Affiliations:** ^1^Lipid Metabolism and Cardiometabolic Disease Laboratory, Baker Heart and Diabetes Institute, Melbourne, VIC, Australia; ^2^Molecular Metabolism and Ageing Laboratory, Baker Heart and Diabetes Institute, Melbourne, VIC, Australia; ^3^Baker Department of Cardiometabolic Disease, University of Melbourne, Parkville, VIC, Australia; ^4^Central Clinical School, Department of Medicine, Monash University, Melbourne, VIC, Australia

**Keywords:** antisense oligonucleotides, lipid metabolism, obesity, fatty liver disease, metabolism

## Abstract

Synthetic oligonucleotide technologies are DNA or RNA based molecular compounds that are utilized to disrupt gene transcription or translation in target tissues or cells. Optimally, oligonucleotides are 10–30 base pairs in length, and mediate target gene suppression through directed sequence homology with messenger RNA (mRNA), leading to mRNA degradation. Examples of specific oligonucleotide technologies include antisense oligonucleotides (ASO), short hairpin RNAs (shRNA), and small interfering RNAs (siRNA). *In vitro* and *in vivo* studies that model obesity related disorders have demonstrated that oligonucleotide technologies can be implemented to improve the metabolism of cells and tissues, exemplified by improvements in fat utilization and hepatic insulin signaling, respectively. Oligonucleotide therapy has also been associated with reductions in lipid accumulation in both the liver and adipose tissue in models of diet-induced obesity. Recent advances in oligonucleotide technologies include the addition of chemical modifications such as N-acetylgalactosamine (GalNAc) conjugates that have been successful at achieving affinity for the liver, in turn improving specificity, and thus reducing off target effects. However, some challenges are still yet to be overcome relating to hepatic injury and off-target effects that have been reported with some compounds, including ASOs. In summary, oligonucleotide-based therapies are an effective tool to elucidate mechanistic insights into metabolic pathways and provide an attractive avenue for translational research into the clinic.

## Introduction

Since the discovery and isolation of DNA in the mid-20th century, the central dogma of molecular biology has been the unidirectional transcription of DNA to RNA, followed by translation into protein (Crick, 1970). Over time, our understanding of this process has been further refined to reflect additional and more nuanced molecular mechanisms that influence these processes. Examples include the discovery of exons and introns, DNA CpG methylation, histone acetylation, non-coding RNAs, response and repressor elements, and alternative splice sites, to name a few. These greater mechanistic insights have enabled the development of a class of therapeutics broadly known as synthetic oligonucleotides or simply, oligo therapies. Synthetic oligonucleotide agents are short sequences of chemically modified DNA or RNA nucleotides that are utilized to alter transcript abundance or translation in target tissues or specific cell populations. Pertinent examples of these molecular tools include antisense oligonucleotides (ASOs), short interfering RNA (siRNA), and short hairpin RNA (shRNA). Collectively, these oligo technologies have been successfully utilized as molecular research tools as well as therapeutic agents in cellular, preclinical, and clinical studies. Herein, we will focus on the utilization of ASOs in the setting of obesity and fatty liver disease as well as discuss the current limitations and challenges encountered with their usage for such a purpose.

## Obesity and Non-Alcoholic Fatty Liver Disease

Based on current global trends in the rate of obesity, approximately 50% of the world will be overweight or obese by 2030 (Smith and Smith, 2016). Obesity is defined clinically by a BMI over 30 kg/m^2^ (Jensen et al., 2014) and is metabolically characterized by the excessive expansion of adipose tissue. White adipose tissue (WAT) is the primary storage site for lipid. However, once saturated, WAT becomes ineffective at storing lipids, resulting in adipocyte dysregulation, systemic dyslipidemia, and deposition of lipids in non-adipose tissues, promoting lipotoxicity. Indeed, these perturbations to lipid homeostasis drive a chronic low-grade inflammatory milieu both in adipocytes themselves as well as in numerous metabolic tissues including the liver (Calder et al., 2011). Consequently, this can promote the development of insulin resistance (Eckel et al., 2005; McQuaid et al., 2011), a significant risk factor for metabolic syndrome. A major clinical consequence of chronic obesity is the development of non-alcoholic fatty liver disease (NAFLD). The links between adipose tissue and the development of NAFLD are well described in both preclincial and clinical studies, where associations have been demonstrated between NAFLD and unhealthy and dysfunctional adipose tissue, including inflammation, insulin resistance, and enhanced collagen synthesis, known as fibrogenesis (du Plessis et al., 2015; Beals et al., 2021). NAFLD is characterized by the accumulation of ectopic lipid in the liver, predominantly triacyclglycerols, known as hepatic steatosis. Simple steatosis and early stage NAFLD can often be a benign condition for a significant period of time, however, can progress rapidly without obvious signs or symptoms (Vernon et al., 2011). Left untreated, NAFLD can progress to a more pathological state in a subset of individuals, known as non-alcoholic steatohepatitis (NASH).

A well established metabolic defect that contributes to dyslipidemia in the setting of high dietary fat intake, is the upregulation of the *de novo* lipogenesis (DNL) pathway (Kawano and Cohen, 2013). The DNL pathway is responsible for the endogenous production of fatty acids (FAs) from acetyl-CoA substrates. These FAs can then be incorporated into numerous lipid species, including sphingolipids, glycerophospholipids, and neutral lipids such as triacylglycerols and cholesterol esters. Increased flux through the DNL pathway can result in the production and accumulation of toxic lipid species, compounding the already lipotoxic cellular environment (Kawano and Cohen, 2013). Obesity and its associated co-morbidities present significant challenges to the healthcare system, highlighting an unmet clinical need for therapeutic interventions to treat these conditions. Current treatment strategies for reducing obesity are met with significant challenges including poor long-term compliance with lifestyle intervention, and several unwanted side effects associated with pharmacological treatments. Thus, the use of oligo therapeutics such as ASOs provides an attractive alternate approach, particularly given their amenability to chemical modification and broad therapeutic treatment window, as discussed below.

## Antisense Oligonucleotides

Antisense oligonucleotides are short, single stranded nucleic acid sequences that are between 10 and 30 base pairs in length (Stein, 2001). ASOs are typically administered *via* injection into either the subcutaneous adipose tissue or peritoneal cavity for *in vivo* preclinical studies. Upon administration, they have a broad tissue distribution and bioavailability, although they primarily accumulate in the liver (first pass metabolism) and in the kidney (excretion; Geary et al., 2015). Notably, given that ASOs do not cross the blood brain barrier, intrathecal injection is required to target a gene of interest (GOI) in the central nervous system (Geary et al., 2015). ASOs are internalized from the systemic circulation *via* endocytosis, however, this process can be augmented substantially *via* the addition of chemical modifications, such as those described below. Upon entry to the cell, ASOs can be deposited into the cytoplasm or sequestered to the nucleus. In both instances, ASOs are designed to bind *via* Watson and Crick hybridization, to a complementary messenger RNA (mRNA) sequence. The resulting mRNA/ASO heteroduplex is then targeted by RNase H1, which mediates their decay (Bennett et al., 2017). RNase H1-dependent degradation results in a reduction in functional mRNA abundance and thus translation of the target mRNA. Current generation ASOs employ a gapmer configuration, an engineered design element whereby the central ASO sequence is homologous to the target sequence and is flanked by stabilizing nucleotides to improve longevity of the ASO. Beyond traditional antisense activity, ASOs can function as an exon skipping agent, whereby ASOs mediate the removal of a mutant or deleterious exon sequence in pre-mRNA that can enable partial rescue of protein function. For example, in Duchenne Muscular Dystrophy (DMD), a hereditary muscle wasting disorder driven primarily by loss of the protein dystrophin, researchers have utilized ASOs to partially restore protein expression of dystrophin (Lim et al., 2017). Briefly, individuals with DMD often carry mutations that disrupt or modify the open-reading frame on exon 51 leading to improper dystrophin protein production (Aartsma-Rus et al., 2009). ASOs directed against exon 51 have thus been shown to clinically improve locomotion in these individuals (Lim et al., 2017).

Considerable work has gone into improving the pharmacokinetics and pharmacodynamics of ASOs. This has been driven by both the need to address the inherent issue of “naked” DNA or RNA degradation, mediated by ubiquitously expressed endonucleases in mammalian organisms, as well to limit off target tissue expression and toxicity. Structural modifications to “native” or unmodified ASOs can be broadly characterized into three distinct groups: nucleic acid modifications (NAMs), backbone modifications (BMs), and macromolecule conjugations. The most common NAMs include locked nucleic acid (LNA) and 2′,4′-constrained 2′-*O*-ethyl bridged nucleic acid (cEt). Substitution for a bridging LNA or cEt nucleic acids improves hybridization and mismatch discrimination for a given sequence (Seth et al., 2010). Common BMs include phosphorothioate (PS) and 2′-O-methoxyethyl (2′-MOE). These backbone modifications increase stability by inhibiting endonuclease degradation of the ASO, although 2′-MOE ASOs have been shown to reduce the affinity for RNase H1 activity (Cook, 1993; Roberts et al., 2020). Recently, Anderson et al. (2021) suggested that next generation ASO chemistries utilizing mesyl-phosphoramidate linkages may address limitations with current generation ASOs. Lastly, a recent addition to the ASO chemistry arsenal is the conjugation of N-acetylgalactosamine (GalNAc) with a native or modified ASO. The GalNAc moiety has previously been shown to facilitate the delivery of non-glycoproteins to the liver (Rogers and Kornfeld, 1971). More recently, it was demonstrated that GalNAc moiety has high affinity for the asialoglycoprotein receptor (ASGPR), which is expressed almost exclusively on hepatocytes (Ashwell and Harford, 1982). The addition of the GalNAc conjugate to ASOs improves their affinity for the liver by ~10-fold compared to native ASOs, while maintaining a toxicity profile that is comparative to native ASOs, as assessed by liver function tests such as plasma alanine transaminase (ALT) levels (Prakash et al., 2014). Currently, published data demonstrating the generation of ASOs that have engineered affinity for adipocytes is lacking.

## UTILIZATION OF ASOs IN THE SETTING OF OBESITY AND NON-ALCOHOLIC FATTY LIVER DISEASE

**Figure 1:**
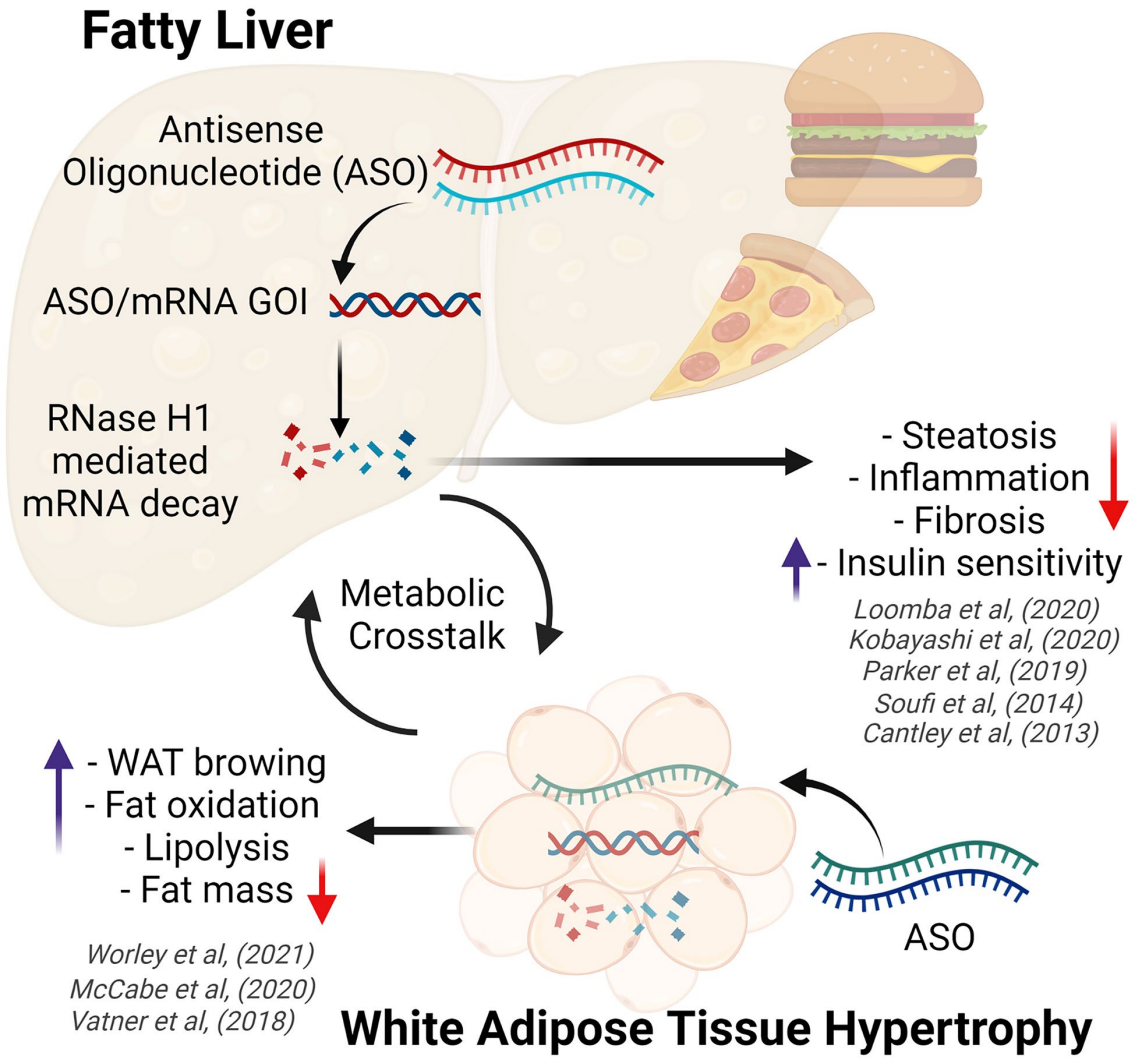
Diagram demonstrating the effects of obesity on white adipose tissue and the liver (fatty liver disease), highlighting key metabolic benefits conferred by antisense oligonucleotide therapy. ASO, antisense oligonucleotides; GOI, gene of interest; WAT, white adipose tissue. Created with BioRender.com.

A greater understanding of the key regulators and pathogenic processes involved in the development and progression of obesity and NAFLD, and ways in which to target them with ASOs has progressed over time. Early ASO studies focused on targeting components of the DNL pathway such as stearoyl–CoA desaturase-1 (SCD-1; Brown et al., 2008). Although *Scd-1*-ASO administration was associated with an attenuation of diet-induced obesity, increased atherosclerotic lesion deposition was observed in these mice compared to control-ASO treated mice. However, targeting other components of the DNL pathway and alternate lipid regulators has yielded more beneficial metabolic effects, as outlined below. Indeed, ASOs have been utilized to improve a range of common pathogenic features associated with obesity and NAFLD, including the dysregulation of lipid metabolism (Yu et al., 2008), lipid droplet dysfunction (Imai et al., 2012; Langhi et al., 2017), improper hepatic lipid partitioning (Nuñez-Durán et al., 2018; Cansby et al., 2019; Caputo et al., 2021), tissue remodeling and fibrosis (Yu et al., 2013; Kobayashi et al., 2020), and adipokine flux (Tan et al., 2015). Here, we have focused on a few select ASO studies that demonstrate effects on obesity and fatty liver disease in preclinical models (Figure 1). These examples describe the utilization of unconjugated (native) ASOs, which lack tissue specificity and thus have broad tissue distribution, silencing target gene expression in multiple tissues including in liver and adipose tissue.

Recently, our group utilized a native ASO to validate a novel regulator of hepatic lipid metabolism (Parker et al., 2019). Silencing of this protein, proteasome 26S subunit non-ATPase 9 (PSMD9), was associated with numerous beneficial metabolic effects. Specifically, we demonstrated, in two mouse strains (C57BL/6J and DBA/2J) fed a western diet, that *Psmd9*-ASO treated mice exhibited a significant reduction in liver steatosis compared to their control-ASO treated counterparts, as indicated by reduced hepatic lipid deposition, specifically diacylglycerol and triacylglycerol species, and hepatocyte ballooning as assessed by histology. Plasma lipid abundance was also significantly reduced, suggesting that overall lipid burden of *Psmd9*-ASO treated mice was attenuated. The significant downregulation of key proteins of the DNL pathway in the livers of *Psmd9*-ASO mice likely contributed to this effect. This is of therapeutic importance given that the DNL pathway is significantly upregulated in individuals with obesity and NAFLD (Diraison et al., 2003). However, greater mechanistic and physiological insight into the role of PSMD9 in the setting of NAFLD is required.

Another example of the use of ASOs in the setting of metabolic syndrome is the targeting of angiopoietin-like 8 (ANGPTL8). ANGPTL8 regulates the expression of lipoprotein lipase (LPL), a protein critical for the lipolysis and trafficking of lipids in mammalian systems (Quagliarini et al., 2012). Mice received weekly intraperitoneal injections of *Angptl8*-ASO, with concurrent feeding of a high fat diet for 3 weeks. *Angptl8*-ASO mice exhibited a significant reduction in liver steatosis and improved insulin sensitivity compared to control-ASO treated mice (Vatner et al., 2018). Hepatic injury was not assessed in mice, but in a cohort of *Angptl8*-ASO treated rats, which also exhibited reduced hepatic steatosis and improved insulin sensitivity, no increase in plasma aspartate transaminase (AST) or ALT levels was observed, a well known side effect of ASO administration (Burel et al., 2016), which is discussed further below. This short study highlights the effectiveness of *Angptl8*-ASO to improve key metabolic readouts that support the maintenance of lipid homeostasis and combat diet-induced NAFLD. However, given the chronic nature of NAFLD, longer term preclinical studies would be required to assess the effectiveness of *Angptl8*-ASO in this setting to understand its capacity to prevent subsequent conditions such as NASH.

Monoacylglycerol acyltransferase 1 (MGAT1), encoded by *Mogat1*, catalyzes the formation of mono- to diacylglycerols, in the lipid synthesis pathway. Inhibition of MGAT1 expression was previously demonstrated to attenuate lipid accumulation induced by oleic acid treatment in HepG2 cells (Yu et al., 2015). Administration of *Mogat1*-ASOs to *ob/ob* mice and wild type mice fed a high fat diet was associated with improved glucose tolerance in the absence of a reduction in hepatic diacylglycerol or triacylglycerol abundance compared to control-ASO treated mice (Hall et al., 2014). Enhanced hepatic insulin signaling was also observed in DIO mice treated with *Mogat1*-ASO, however, no change in body weight was seen. Soufi et al. (2014) administered ASOs against *Mogat1* to C57BL/6J mice fed a modified high fat, high cholesterol diet, that promotes hepatic injury and inflammation. They also demonstrated that mice exhibited improved glucose tolerance and hepatic insulin signaling (Soufi et al., 2014). Moreover, *Mogat1*-ASO treated mice exhibited a significant reduction in weight gain compared to control-ASO treated mice. Epididymal WAT mass and hepatic triacylglycerol abundance were also significantly reduced in *Mogat1*-ASO mice, although no improvements in liver inflammation or injury were observed, suggesting that targeting *Mogat1* may provide metabolic benefit in early liver disease. Further studies by this group examined the effect of *Mogat1*-ASOs in *Mogat1* whole body knockout mice (MOKO) fed a high fat diet and demonstrated improved glucose tolerance in these mice (Lutkewitte et al., 2021). These findings recapitulate their abovementioned findings with *Mogat1*-ASO, however, they suggest that *Mogat1*-ASOs may improve glucose homeostasis independent of *Mogat1* knockdown. A significant induction of type I interferon responsive genes, *Oasl1*, *Ifit1*, and *Ifit2* was seen in *Mogat1*-ASO treated MOKO mice, with a similar trend observed in *Mogat1*-ASO treated wild type mice, consistent with the findings of McCabe et al. (2020), as discussed below. However, co-administration of a type I interferon alpha/beta receptor-1 (IFNAR1) neutralizing antibody to wild type did not impact on the improvements in glucose tolerance seen with the *Mogat1*-ASO, suggesting that this pathway was not responsible for the effect on glucose handling.

Diacylglycerol O-Acyltransferase 2 (DGAT2) catalyzes the final step in the triacylglycerol synthesis pathway. Targeting of DGAT2 in preclinical dietary models has yielded mixed results. Researchers have observed improvements in hepatic steatosis with a marked reduction in liver triacylglycerol content with *Dgat2*-ASO administration in mice fed a high fat diet (Yu et al., 2005). Likewise, Yamaguchi et al. (2007) observed that in *db/db* mice fed a methionine choline deficient diet, a model of NASH, *Dgat2*-ASO administration was associated with improved liver steatosis. Despite this, the authors reported an increase in hepatic free FAs, fibrosis, and an upregulation of oxidative stress markers in *Dgat2*-ASO treated *db/db* mice. *Dgat2*-ASO administration was also associated with elevated ALT levels, indicating hepatic injury. One possible explanation for these discrepancies is a difference in the severity of the diet and genetic models utilized. More severe models can be associated with irreversible damage to cellular machinery and metabolic processes rendering treatments such as *Dgat2*-ASO, which appears to impact on steatosis alone, ineffective. These contrasting studies highlight a key consideration when developing ASOs for therapeutic utility, as timing of ASO administration is likely to be a critical factor impacting on their efficacy.

Overall, these preclinical studies establish strong evidence that targeting lipid regulatory pathways in the liver with ASOs can have beneficial effects on hepatic steatosis and subsequent pathological processes. However, the variability and limitations of currently available preclinical models and the severity of their phenotype, need to be considered in the context of the translational pipeline.

## Challenges and Limitations in ASO Studies

Given that the liver is a major site of ASO uptake, ASO-mediated hepatic toxicity remains a significant challenge (Burel et al., 2016). Many studies that have engaged ASO technology to reduce target gene expression, have reported on the impact of these therapeutics on liver function through the measurement of ALT, AST, and/or gamma-glutamyltransferase (GGT). Elevated levels of these liver enzymes can vary widely in response to ASO treatment. For example, ASOs have previously been shown to induce a severe inflammatory response in some cases (Burel et al., 2012). However, plasma levels of ALT and AST can be difficult to interpret as these markers can be elevated in models of general liver dysfunction, thus readouts can be confounded by improvements in liver function due to ASO-mediated actions, as seen with ASOs targeting *Stk25* (Cansby et al., 2019). Nephrotoxicity has also been observed with *in vivo* LNA-ASO usage (Moisan et al., 2017). Using a human kidney cell model, researchers demonstrated that epidermal growth factor (EGF) was an effective *in vitro* biomarker of LNA-ASO induced kidney injury.

Off-target effects present a substantial challenge to ASO based approaches, as highlighted by studies that administered *Mogat1*-ASOs (Lutkewitte et al., 2021). Induction of the type I interferon response observed in this study, was similarly reported in a recent publication by McCabe et al. (2020). Here, authors used multiple ASOs sequences to silence tetratricopeptide repeat protein 39B (TTC39B) in mice, resulting in protection against diet-induced obesity. Unexpectedly, *Ttc39b*-ASOs also elicited a type I interferon response in gonadal WAT (gWAT), likely originating from resident adipose tissue macrophages. The authors demonstrated independently *via* RNAseq and quantitative PCR that the genes *Ifit1*, *Ifit3*, and *Oasl1* were significantly elevated. In contrast to the *Mogat1*-ASO studies by Lutkewitte et al. (2021), McCabe et al. (2020) demonstrated that the effect on body weight gain was abolished in IFNAR1 knockout mice, indicating that this was an IFNAR1-dependent effect. However, when authors used liver-targeted GalNAc-ASOs, targeting hepatocytes, this response was not observed. An important caveat to these studies is whether these finding are due to different ASO chemistries, the lack of targeting in adipose tissue with GalNAc-ASOs, or whether sequence specificity contributes to this phenomenon, the latter of which has been explored in some detail. Indeed, the evidence for sequence specific toxicity in ASOs arises from studies in mice utilizing over 70 distinct ASOs sequences (Burdick et al., 2014). Researchers identified 58 permutations of 2–5 base pairs that were hepatotoxic (Burdick et al., 2014). Specifically, TGC and TCC motifs were shown to be strongly associated with toxicity as assessed by AST and ALT levels, and this was attributed to aberrant ASO binding to critical hepatic proteins that regulate cell cycling and apoptosis (Burdick et al., 2014).

Lastly, practical considerations are also worth noting with regard to oligonucleotide therapy for conditions such as obesity. For example, ASOs are administered *via* injection, which can be undesirable for patients that experience needle phobia, a condition that affects close to 30% of the general adult population (McLenon and Rogers, 2019). There are also cost considerations. ASO therapies generally cost significantly more than oral medications. For example, Spinraza®, an ASO used in the treatment of spinal muscular atrophy, had a price indication of US$750,000 for the first year of treatment in 2016 (Maharshi and Hasan, 2017). These price regimens may therefore preclude the widespread use of ASOs. There may also be resistance from patients due to the perceived risk of genetic manipulation as seen with COVID-19 mRNA vaccines (Hotez et al., 2021). These downsides should, however, be balanced against the potential benefits of ASO therapeutics, which have a long-term efficacy upward of several months in humans, and thus optimized treatment might require injections only 2–3 times per year, significantly cutting costs and negating the need for daily treatment.

## Future Perspectives

Antisense oligonucleotides represent a promising avenue as selective agents to ameliorate the adverse effects associated with the dysregulation of lipid homeostasis in the setting of obesity and its complications. Equally though, challenges exist in designing long-term, high-quality studies to assess their effectiveness in preclinical models that more accurately exhibit key metabolic features and reflect the disease progression observed in obesity and NAFLD. A greater understanding of the off-target effects associated with specific ASO sequences will be required. Finally, regression studies are critical to assessing the ability of ASOs to reverse established disease. This is particularly relevant in the clinical setting, as it represents an important steppingstone for the translation of ASOs to the clinic.

## Author Contributions

MK, BD, and AC contributed to conception and design of the review and wrote and edited sections of the manuscript. MK wrote the first draft of the manuscript. All authors contributed to manuscript revision, read, and approved the submitted version.

## Funding

This work was supported in part by the Victorian State Government OIS Program to the Baker Heart and Diabetes Institute. BD and AC have received support from the National Heart Foundation of Australia Future Leader Fellowship scheme (101789 and 105631, respectively).

## Conflict of Interest

BD and AC have patents for the use of ASOs in obesity and fatty liver and have material transfer agreements with Ionis Pharmaceuticals relating to projects that use ASO technology.

The remaining authors declare that the research was conducted in the absence of any commercial or financial relationships that could be construed as a potential conflict of interest.

## Publisher’s Note

All claims expressed in this article are solely those of the authors and do not necessarily represent those of their affiliated organizations, or those of the publisher, the editors and the reviewers. Any product that may be evaluated in this article, or claim that may be made by its manufacturer, is not guaranteed or endorsed by the publisher.
